# Electrodeposition of tin on Nafion-bonded carbon black as an active catalyst layer for efficient electroreduction of CO_2_ to formic acid

**DOI:** 10.1038/s41598-017-14233-y

**Published:** 2017-10-20

**Authors:** Qinian Wang, Xinqi Wang, Chao Wu, Yuanyuan Cheng, Qingye Sun, Heng Dong, Hongbing Yu

**Affiliations:** 1 College of Resources and Environmental Engineering, Anhui University, Hefei, Anhui 230601 China; 2 College of Life Sciences, Anhui University, Hefei, Anhui 230601 China; 3 College of Environmental Science and Engineering, Nankai University, Tianjin, 300071 China

## Abstract

Electroreduction of CO_2_ to formic acid (ERCF) based on gas diffusion electrodes (GDEs) has been considered as a promising method to convert CO_2_ into value-added chemicals. However, current GDEs for ERCF suffer from low efficiency of electron transfer. In this work, a novel Sn-based gas diffusion electrode (ESGDE) is prepared by electrodepositing Sn on Nafion-bonded carbon black as catalyst layer to enhance electron transfer and thus the efficiency of ERCF. The highest Faraday efficiency (73.01 ± 3.42%), current density (34.21 ± 1.14 mA cm^−2^) and production rate (1772.81 ± 59.08 μmol m^−2^ s^−1^) of formic acid are obtained by using the ESGDE with electrodeposition time of 90 s in 0.5 M KHCO_3_ solution, which are one of the highest values obtained from Sn-based gas diffusion electrodes under similar conditions. The notable efficiency of ERCF achieved here should be attributed to the enhancement in the reactants transfer as well as the three-dimensional reaction zone. This work will be helpful for the industrial application of GDEs in EFCF.

## Introduction

Increased emission of carbon dioxide (CO_2_) in the atmosphere emitted from anthropogenic activities is thought to have a significant impact on the climate change^1–3^. Therefore, decreasing CO_2_ atmospheric concentration has become an urgent problem^4–6^. Electroreduction of CO_2_ can not only reduce emission load, but also retrieve a variety of valuable products such as formic acid (HCOOH), methane (CH_4_), carbon monoxide (CO), methanol (CH_3_OH), ethylene (C_2_H_4_) and so on^7–12^. Among various reduction products from electroreduction of CO_2_, formic acid is one of the highest value-added chemicals, which has a broad market and wide application range in dyeing, food additives, and pharmaceutical industries^13^. Plenty of researchers have declared that electroreduction of CO_2_ to formic acid (ERCF) as industrial application is economically feasible. The economic feasibility of the ERCF process depends strongly on the electrode, involving its electrochemical performance and the cost^13–16^.

Tin (Sn) is one of the state-of-the-art non-noble metal catalysts for ERCF^17^. In recent years, a number of novel Sn-based catalysts, such as nanostructured Sn catalysts^18,19^, and supported Sn catalysts^20^, have been exploited to improve the selectivity and throughput of the ERCF process. However, relatively fewer efforts have focused on the study of the structure and/or composition of the associated electrodes, which are the key to maximize the activity of the catalysts on the electrodes. To date, the most common electrodes for ERCF are metal foil^21^, metal plate^15,16^ and gas diffusion electrode (GDE)^22,23^. By contrast, GDE can improve mass transport of CO_2_, which is particularly important for ERCF given the low solubility of CO_2_ in water^23^. GDE is a three-dimensional composite electrode, which usually consists of a catalyst layer (CL) and a gas diffusion layer (GDL)^17,23^. Recently, several literatures reported the use of Sn-based GDE for formic acid formation^22,23^ while several works mentioned the use of Cu_2_O, Cu_2_O/ZnO, Cu and Cu metal-organic porous materials as catalysts on GDE for alcohols and hydrocarbons formation^4,24–27^. In addition, Ag-based GDE had been used for carbon monoxide formation^28^. The traditional method for fabricating the GDE mentioned above is spraying a catalyst ink consisting of catalyst powder and Nafion binder onto a GDL (i.e. carbon paper)^17,23,28^. Although the use of GDE led to significant improvement in the performance of electroreduction of CO_2_, there are several problems needed to be solved for its industrial application. For example, the electrode cost is high (ca. 1050 $ m^−2^)^29,30^. Moreover, the sufficient reactants (electron, proton and CO_2_) transfer is essential to an efficient ERCF^28,29^. However, due to the nonconductivity of Nafion, the electron transfer between the CL (i.e. merely consists of catalyst and Nafion) and GDL, as well as that within the CL (i.e. merely consists of catalyst and Nafion) are blocked, which restrain the efficiency of ERCF on the traditional GDE. Therefore, it is necessary to develop a new GDE to solve these problems for the industrial application of GDE in EFCF.

**Figure 1:**
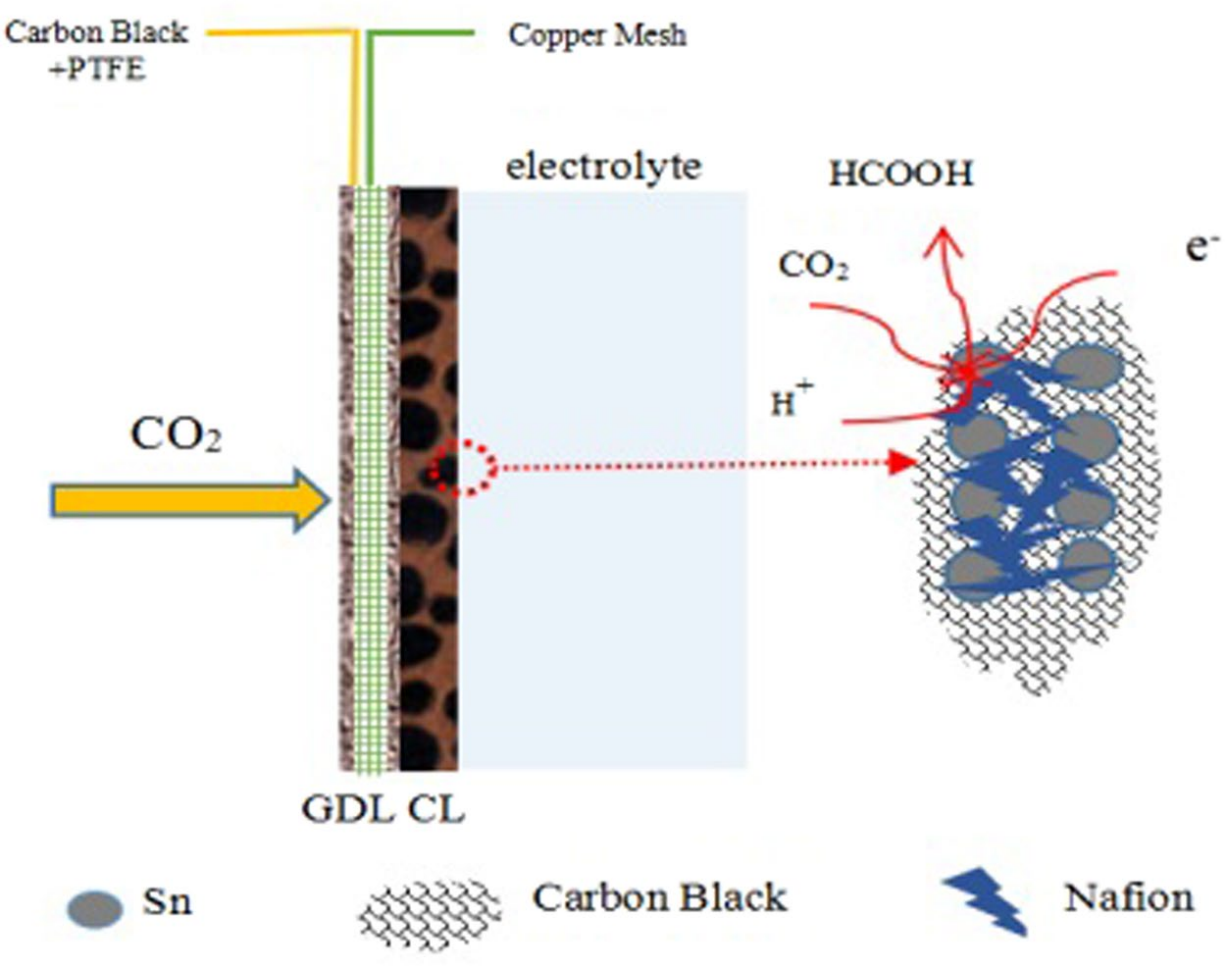
Schematic diagram of the ESGDE.

In our previous work, an economic Sn-based GDE (SGDE) with a GDL consisting of carbon black, polytetrafluoroethylene (PTFE) and copper mesh has been developed for ERCF^29^. It has been evidenced that the performance of the SGDE is equal to that of Sn-based GDE with a carbon paper GDL. While, owing to the low-cost of carbon black, PTFE and copper mesh in the GDL (ca. 30 $ m^−2^) of the SGDE, the cost of the SGDE (ca. 80 $ m^−2^) is 92% lower than that of Sn-based GDE with a carbon paper GDL (ca. 1050 $ m^−2^)^29^. Therefore, the primary objective of this work is to further enhance electron transfer and thus the efficiency of ERCF on the SGDE. For this purpose, a novel CL made by electrodeposition Sn on Nafion-bonded carbon black was dispersed on the GDL of the SGDE and the fabricated electrode is called ESGDE (see Fig. 1). A detailed physical characterization for the ESGDE were carried out by scanning electron microscopy (SEM), transmission electron microscopy (TEM), X-ray diffraction (XRD) and X-ray photoelectron spectroscopy (XPS). The electrochemical performance of the ESGDE for ERCF was investigated by cyclic voltammetry (CV), electrochemical impedance spectroscopy (EIS) and constant potential electrolysis. The effect of electrodeposition time on the performance of the ESGDE for ERCF was also studied.

## Results

### ESGDE characterization

**Figure 2:**
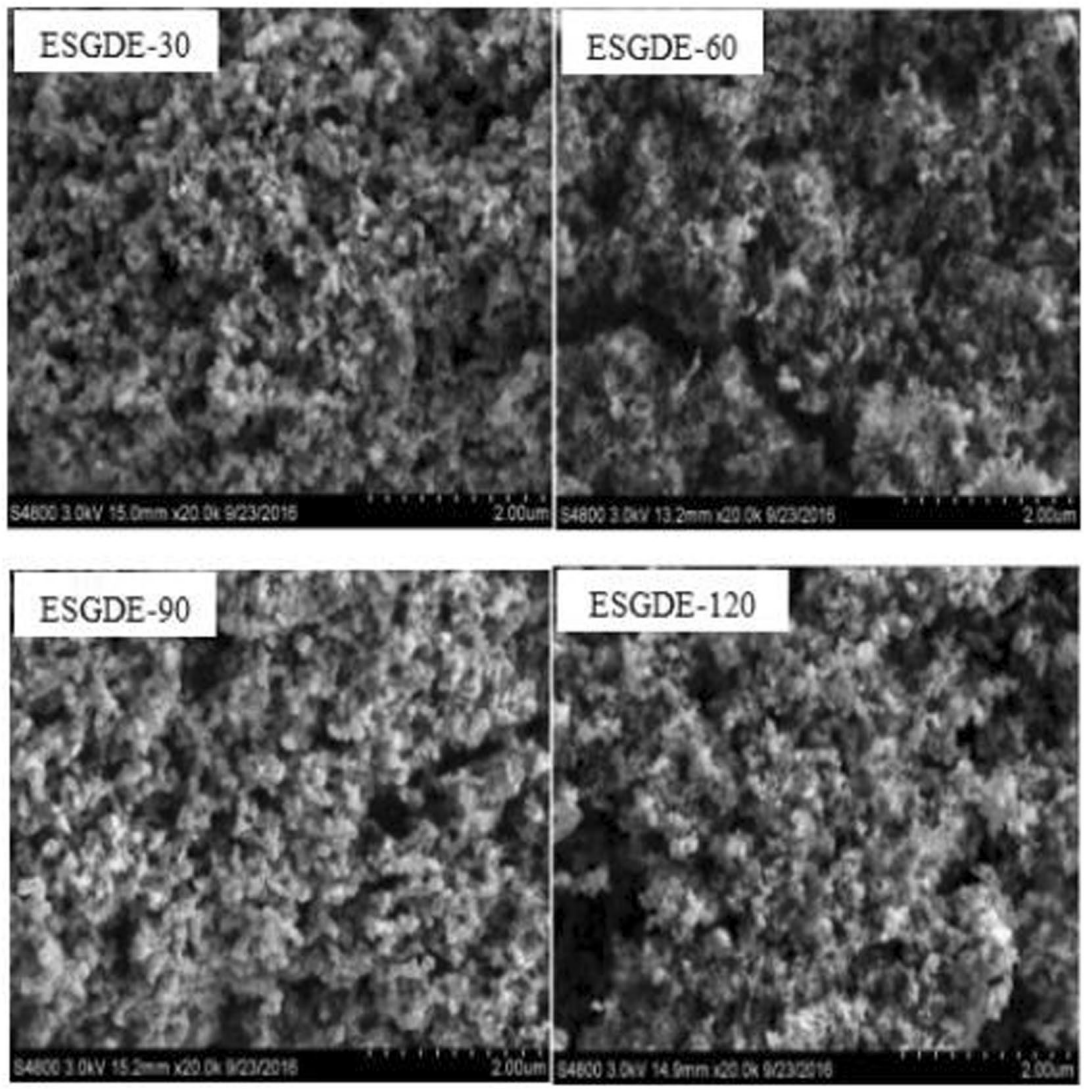
SEM of the catalyst layer in the ESGDEs with different electrodeposition time at magnification of 20 K.

**Figure 3:**
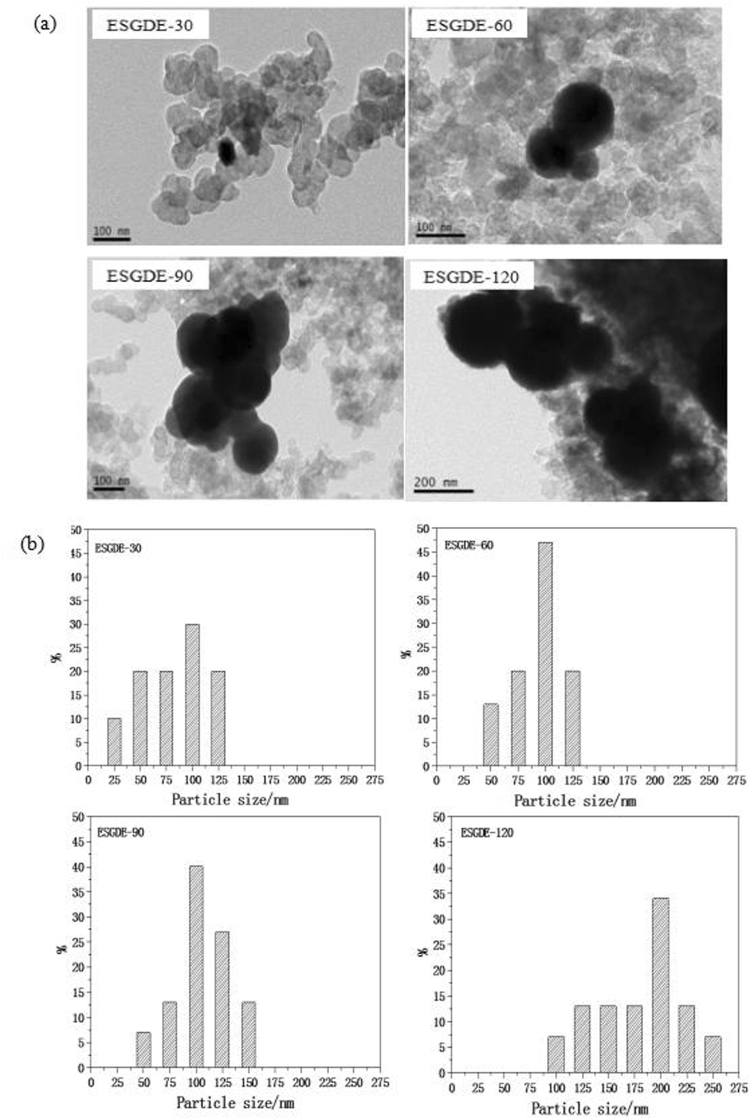
(**a**) TEM of the Sn particles in the ESGDEs with different electrodeposition time. (**b**) Particle size distributions of the Sn particles in the ESGDEs with different electrodeposition time. Distribution plots were calculated based on several TEM images.

SEM images for the CL and the TEM images for the Sn catalyst in the ESGDEs with different electrodeposition time are displayed in Fig. 2 and Fig. 3a. It can be seen that each electrode has abundant porous structure made of carbon black and the Sn particles are randomly distributed on the surface of the carbon black. With the increase of electrodeposition time, the loading amount and average particle size of Sn catalyst increased (Table S1, Fig. 3b). When the electrodeposition time was increased from 30 s to 60 s, the loading amount increased from 0.9 mg cm^−2^ to 1.9 mg cm^−2^ and the average particle size increased from ca. 85 nm to 95 nm. When the electrodeposition time was further increased from 90 s to 120 s, the loading amount increased from 2.6 mg cm^−2^ to 3.4 mg cm^−2^ and the average particle size increased from ca. 105 nm to 180 nm.

**Figure 4:**
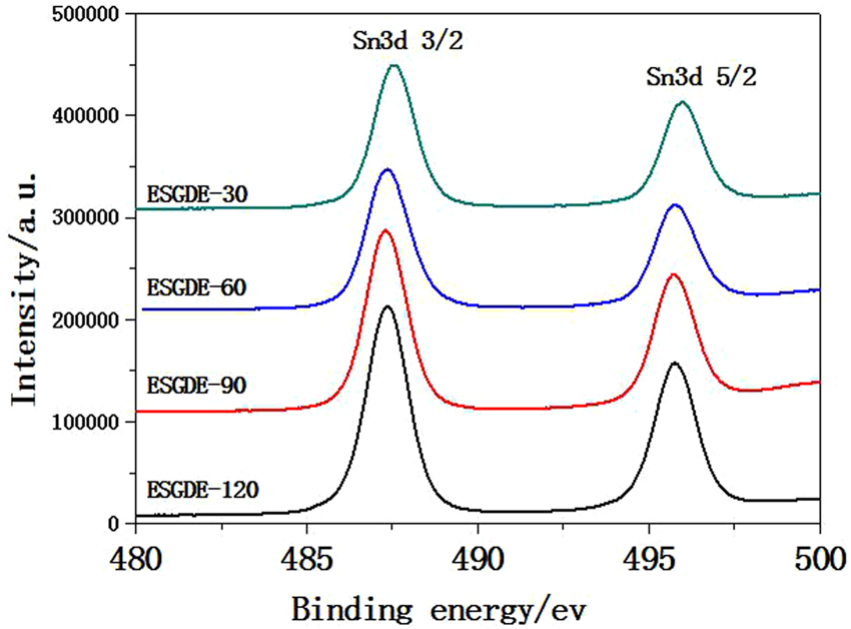
XPS spectrum of Sn3d in the ESGDEs with different electrodeposition time.

The XPS spectrum of Sn3d in the ESGDEs with different electrodeposition time consist of two peaks at the binding energies of ca. 495 eV and 487 eV, which can be assigned to Sn3d_3/2_ and Sn3d_5/2_, respectively (Fig. 4). These binding energies are corresponding to the XPS spectrum for oxidized tin (SnO_x_), because Sn oxidation to form SnO_x_ is a highly spontaneous reaction^31^. The XRD pattern of the ESGDEs with different electrodeposition time as shown in Figure S1 (see supplementary information) clearly displayed the phase of Sn (JCPDS Card no. 65-7657). The different results of XPS and XRD probably because XPS can only parse the oxidation states of Sn in the outer layer of ca. 10 nm of the specimen^32^. Therefore the outer and inner layer of the Sn catalysts on the ESGDEs existed in the forms of SnO_x_ and Sn. The SnO_x_ layer in the outer of the Sn catalyst on the ESGDEs is beneficial for ERCF by restraining hydrogen evolution reaction (HER)^32^.

### Electrochemical measurements

**Figure 5:**
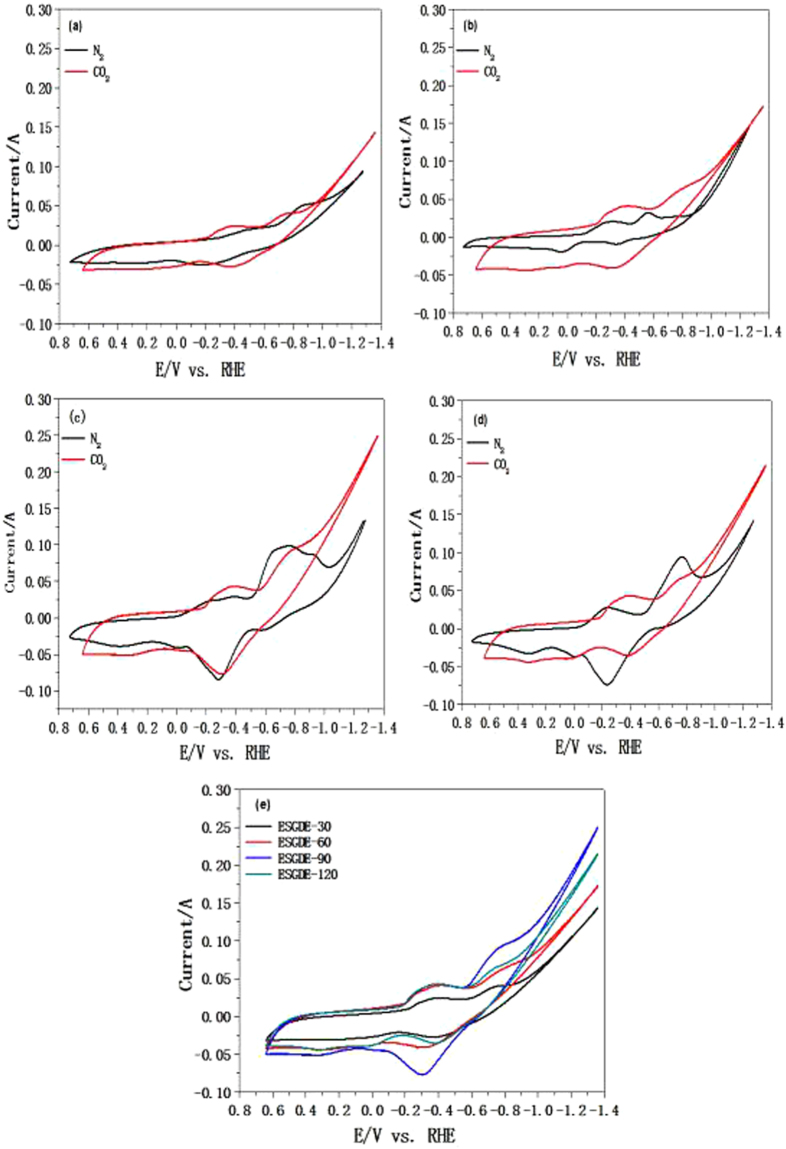
CVs obtained from the ESGDEs with electrodeposition time of 30 s (**a**), 60 s (**b**), 90 s (**c**) and 120 s (**d**) in 0.5 M N_2_-saturated KHCO_3_ solution (pH = 8.92) and CO_2_-saturated KHCO_3_ solution (pH = 7.47), scanning rate: 0.1 V s^−1^. (**e**) Comparison of the CVs obtained from the ESGDEs with different electrodeposition time in 0.5 M CO_2_-saturated KHCO_3_ solution (pH = 7.47).

The electrochemical behavior of the ESGDEs with different electrodeposition time were preliminarily detected by CV in 0.5 M N_2_- and CO_2_- saturated KHCO_3_ solution, respectively (Fig. 5). The pH value of 0.5 M N_2_- and CO_2_- saturated KHCO_3_ solution was 8.92 and 7.47, respectively. As the pH value seriously effect the electrode potentials for ERCF and HER, the electrode potentials were converted to the reversible hydrogen electrode (RHE) scale using E (vs. RHE) = E (vs. Ag/AgCl) + 0.1988 + 0.0591 V × pH^15^. The reduction peaks between ca. −0.2 V vs. RHE and −0.6 V vs. RHE while the oxidation peaks between ca. −0.3 V vs. RHE and 0 V vs. RHE in Fig. 5a–d should be attributed to the reduction and formation of SnO_x_ during the potential scan^15,22^. Won *et al*. recorded the similar reduction peak of SnO_x_ at −0.56 V vs. RHE^18^ while Zhang *et al*. reported the reduction peak of SnO_2_ at a potential of −0.36 V vs. RHE^19^. On the cathodic end of the CVs, rapidly increase in the reduction currents can be seen under both N_2_- and CO_2_- saturated electrolyte in each case. Moreover, the reduction currents in CO_2_-saturated electrolyte are higher than those in N_2_-saturated electrolyte. The rapidly increase reduction currents in N_2_-saturated electrolyte should be caused by HER^15,22^ while those in CO_2_-saturated electrolyte should be caused by ERCF as well as HER^22,33^. This is verified when the constant potential electrolysis tests performed under both N_2_- and CO_2_- saturated electrolyte, which shows that formic acid can only produce in CO_2_- saturated electrolyte. The onset potential of ERCF at the ESGDEs is ca. −0.5 V vs. RHE, which is consistent with the reported values for Sn electrodes^18,19^. Figure 5e compared the CVs obtained at the ESGDEs in CO_2_-saturated electrolyte, it can be seen that the reduction current increased when the electrodeposition time increased from 30 s to 90 s but it decreased when the electrodeposition time further increased to 120 s.

**Figure 6:**
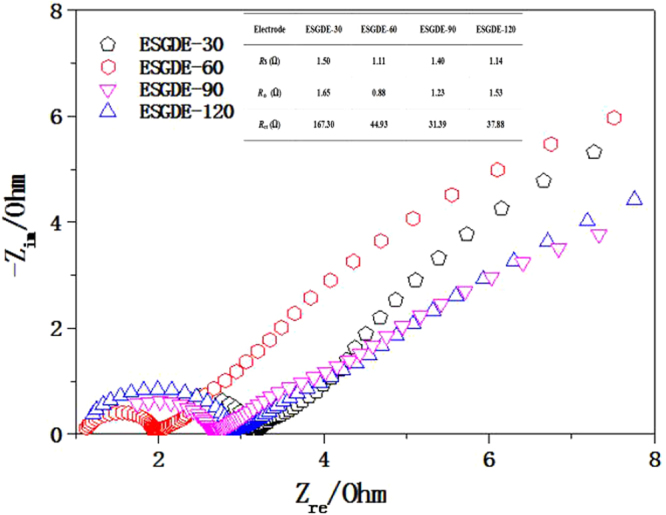
Nyquist plots of the ESGDEs in 0.5 M CO_2_-saturated KHCO_3_ solution (pH = 7.47). Inset figure: simulated data of equivalent circuits for the Nyquist plots of impedance. *R*_s_: solution resistance, *R*_Ω_: Ohm resistance, *R*_ct_: charge transfer resistance.

The electrochemical behavior of the ESGDEs with different electrodeposition time were further evaluated by EIS experiments (Fig. 6). The Nyquist plots all consisted of two semicircles and were modeled by the same equivalent circuit (Figure S2 in the supplementary information) using a software named ZsimpWin. The simulated data of equivalent circuits for the Nyquist plots of impedance are given in the inset figure in Fig. 6. The solution resistances (*R*s) and Ohmic resistances (*R*_Ω_) values for the ESGDEs with different electrodeposition time were almost similar. Whereas obvious differences were found in the charge transfer resistances (*R*_ct_), which decrease as follows: 167.30 Ω (ESGDE-30) > 44.93 Ω (ESGDE-60) > 37.88 Ω (ESGDE-120) > 31.39 Ω (ESGDE-90). It indicates that extending electrodeposition time in a certain range (<90 s here) can accelerate the reduction reactions (involving HER and ERCF), which is in accord with the CV results mentioned above.

The active surface areas of the ESGDEs with different electrodeposition time were evaluated by measuring the double layer capacitance in N_2_-saturated 0.1 M KHCO_3_ solution (pH = 8.72)^34^. As shown in Figure S3 in the supplementary information, surface CVs were recorded on both the ESGDE without Sn catalyst (noted as ESGDE-0) and the ESGDEs in a potential scope without Faraday process. The capacitance (*C*) is calculated according to the equation *C* = current density/scan rate^34^. The capacitance of the ESGDE-0 is 176 μF cm^−2^, which is in agreement with those results reported previously on capacitance of carbon materials^34^. However, under the same measuring conditions, the capacitance for the ESGDE-30, ESGDE-60, ESGDE-90 and ESGDE-120, is 2402 μF cm^−2^, 3295 μF cm^−2^, 4186 μF cm^−2^ and 2676 μF cm^−2^, respectively. According to their surface capacitances, if the surface area of the ESGDE-0 is 3.14 cm^2^, that of the ESGDE-30, ESGDE-60, ESGDE-90 and ESGDE-120 could be calculated to be 42.85 cm^2^, 58.79 cm^2^, 74.68 cm^2^ and 47.74 cm^2^, respectively. Obviously, the active surface area of the ESGDE increased when the electrodeposition time increased from 30 s to 90 s but it decreased when the electrodeposition time further increased to 120 s.

### ERCF tests

**Figure 7:**
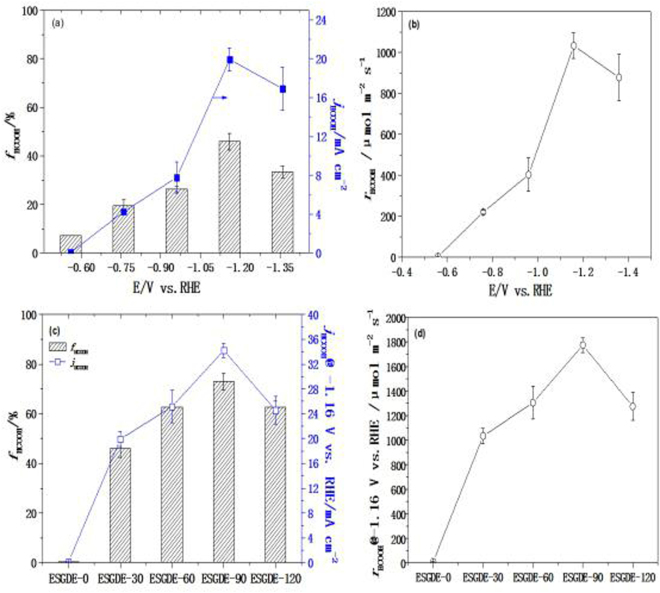
(**a**,**b**) Variations in the *f*_HCOOH_, *j*_HCOOH_ and *r*_HCOOH_ with electrolysis potential in 0.5 M CO_2_-saturated KHCO_3_ solution (pH = 7.47) at the ESGDE-30. (**c**,**d**) ERCF tests results at -1.16 V vs. RHE in 0.5 M CO_2_-saturated KHCO_3_ solution (pH = 7.47) at the ESGDEs with different electrodeposition time and ESGDE-0. Error bars based on three duplicate measurements for each test.

ERCF tests were first performed to investigate the effect of electrolytic potential on the Faraday efficiency (*f*_HCOOH_), current density (*j*_HCOOH_) and production rate (*r*_HCOOH_) of formic acid at the ESGDE-30 in 0.5 M CO_2_-saturated KHCO_3_ solution (pH = 7.47) (Fig. 7a and b). It can be noticed that *f*_HCOOH_ increases when the electrolytic potential shifts from −0.56 V vs. RHE to −1.16 V vs. RHE, but it decreases when the electrolytic potential further shifts to −1.36 V vs. RHE. The maximum *f*_HCOOH_ (45.91 ± 3.42%) is achieved at the electrolytic potential of −1.16 V vs. RHE, which is 5.3 times higher than that of −0.56 V vs. RHE (7.26 ± 0.01%). Similar to *f*_HCOOH_, the maximum *j*_HCOOH_ (19.93 ± 1.21 mA cm^−2^) and *r*_HCOOH_ (1032.80 ± 62.71 μmol m^−2^ s^−1^) are also obtained at the electrolytic potential of −1.16 V vs. RHE, which are 331.1 times and 331.1 times higher than those of −0.56 V vs. RHE (*j*_HCOOH_: 0.06 ± 0.01 mA cm^−2^, *r*_HCOOH_: 3.11 ± 0.52 μmol m^−2^ s^−1^), respectively. Thus, the optimal electrolytic potential for efficient ERCF should be −1.16 V vs. RHE (i.e. −1.8 V vs. Ag/AgCl) in our conditions, which is consistent with those results reported previously for Sn electrodes^13,15,17,35^.

In order to deeply understand the effects of electrodeposition time on the performance of the ESGDE, ERCF tests were carried out at the optimal electrolytic potential of −1.16 V vs. RHE at the ESGDEs with different electrodeposition time in 0.5 M CO_2_-saturated KHCO_3_ solution (pH = 7.47) (Fig. 7c and d). The ERCF experiment was also carried out using the ESGDE without Sn catalyst (noted as ESGDE-0). It can be noticed that *f*_HCOOH_ (0.31 ± 0.05%), *j*_HCOOH_ (0.15 ± 0.03 mA cm^−2^) and *r*_HCOOH_ (7.77 ± 1.55 μmol m^−2^ s^−1^) obtained from the ESGDE-0 were very low, indicating the ESGDE-0 almost has no catalytic activity for ERCF. When the electrodeposition time increases from 30 s to 90 s, *f*_HCOOH_, *j*_HCOOH_ and *r*_HCOOH_ increase. While they decrease when the electrodeposition time further increases to 120 s. The maximum *f*_HCOOH_ (73.01 ± 3.42%), *j*_HCOOH_ (34.21 ± 1.14 mA cm^−2^) and *r*_HCOOH_ (1772.81 ± 59.08 μmol m^−2^ s^−1^) were achieved from the ESGDE-90, which are 59%, 72% and 72% higher than those from the ESGDE-30 (*f*_HCOOH_: 45.91 ± 3.42%; *j*_HCOOH_: 19.93 ± 1.21 mA cm^−2^; *r*_HCOOH_:1032.80 ± 62.71 μmol m^−2^ s^−1^), respectively.

## Discussion

The ERCF (CO_2_ + 2 H^+^  + e^−^ → HCOOH) requires CO_2_, electrons and protons simultaneously^23,29^. However, mass transport limitation of CO_2_ impedes ERCF severely at solid electrode (such as metal foil^21^, metal plate^15,16^) due to the low solubility of CO_2_ in water (ca. 0.033 M). It has been proved that the use of GDE is an effective method to alleviate mass transfer limitation of CO_2_^4,22–28^. When a GDE is used for ERCF, CO_2_, electrons and protons come through the GDL, the CL and the electrolyte-CL interface, respectively^23,29^. The sufficient electron and proton transfer, as well as CO_2_ diffusion are essential to an efficient ERCF^23,29^. In the CL of the traditional Sn-based GDE (i.e. merely consists of catalyst and Nafion), Nafion has dual role for ERCF. For one thing, it can enhance proton transfer in the CL and also allows the integration of the CL with the electrolyte^23,29^, which is favor for ERCF. For the other thing, Nafion can not conduct electron, which damages the electron transfer between the CL and the GDL, as well as the electron transfer within the CL. This leads to a decrease in ERCF. Therefore, the efficiency of ERCF on the traditional Sn-based GDE is much possibly improved by enhancing the electron transfer while the proton transfer and CO_2_ diffusion are sufficient simultaneously. In consideration of that carbon black is porous and conductive, we developed Nafion-bonded carbon black as CL support, anticipating an improvement in the electron transfer via carbon black and in proton transfer through Nafion. Moreover, carbon black can create a number of pores (see Fig. 2), while Nafion allows the integration of pores with the electrolyte, therefore the Nafion-bonded carbon black support designed in the ESGDE played a direct role in accelerating the ERCF via increasing the three-dimensional reaction zone and reactants transfer substantially^23,29^. In addition, Nafion and conductive carbon black contributed many sites with characterization of that electrolyte (SnCl_2_ solution) and electron are easily accessible, which are just the requirement of electrodeposition of Sn reaction (Sn^2+^  + 2e^−^ → Sn). So the Nafion-bonded carbon black support designed in the ESGDE played an indirect role in accelerating the ERCF via promoting Sn catalyst loading. The carbon black loading amount and Nafion fraction in the Nafion-bonded carbon black CL support are the two crucial parameters for the CO_2_ diffusion, electron and proton transfer. For the carbon black loading amount, prior works in proton exchange membrane fuel cells had shown that a loading amount of 1 mg cm^−2^ could form a complete layer on top of the GDL and allow easy diffusional access for the gas to reach the reaction sites^36^. While for the Nafion fraction, a optimized Nafion fraction of 50 wt.% in the CL had been reported in our previous work^29^. Therefore, we chose a carbon black loading amount of 1 mg cm^−2^ and a Nafion fraction of 50 wt.% in this work, which can provide a desirable CO_2_ diffusion, electron and proton conduction for an efficient ERCF.

Electrodeposition is a facile, timesaving and low-cost method to prepare Sn catalyst^37^. The characteristics of the Sn catalyst (e.g., size, loading amount) are determined by several parameters including the electrodeposition time, the applied overpotential, and so on^38^. In this work, the electrodeposition time was studied preliminarily. As shown in Fig. 2 and Fig. 3, it can be seen that the loading amount and average particle size of Sn catalyst increase with the increase of the electrodeposition time. It has been reported that the loading amount and particle size of Sn catalysts have a drastic effect on the activity of ERCF^18,19,29^, which is also can be seen in this work. According to the results of SEM, TEM, CV, EIS, active surface area measurement and constant potential electrolysis, it can be inferred that extending electrodeposition time in a certain range (<90 s here) can increase the loading amount and size of Sn catalyst, resulting in an increase in the active surface area and a decrease in the charge transfer resistance, which can accelerate the ERCF. While if the electrodeposition time is out of that range (e.g. 120 s here), a decrease in the active surface area and an increase in the charge transfer resistance may be caused and restrain the ERCF. Under our experimental conditions, the optimal electrodeposition time is 90 s and the maximum *f*_HCOOH_, *j*_HCOOH_ and *r*_HCOOH_ are 73.01 ± 3.42%, 34.21 ± 1.14 mA cm^−2^ and 1772.81 ± 59.08 μmol m^−2^ s^−1^, respectively. For a proper analysis of the effect of electrodeposition time on the performance of the ESGDEs for ERCF, the *r*_HCOOH_ is normalized by catalyst loading (*r* ^m^_HCOOH_), active surface area (*r* ^s^_HCOOH_) and charge passed through the system (*r* ^q^_HCOOH_). As shown in Table S2 (see supplementary information), it can be seen that *r* ^m^_HCOOH_ and *r* ^s^_HCOOH_ both decreased when the electrodeposition time increase from 30 s to 120 s. While *r* ^q^_HCOOH_ increased when the electrodeposition time increased from 30 s to 90 s but it decreased when the electrodeposition time further increased to 120 s. This results suggest the existence of Sn catalyst agglomeration in the ESGDEs with the increased of the electrodeposition time^4,25^. This is in accord with the TEM results. This results also indicate that improving the dispersion degree of the Sn catalyst on the ESGDEs can further enhance the efficiency of ERCF.

It has been explained above that why the ESGDE-90 can enhance efficiency of ERCF compare to the traditional Sn-based GDE (i.e. SGDE in this work). In order to confirm the advantage of the ESGDE-90 compare to the traditional Sn-based GDE, a control test was carried out. The *f*_HCOOH_ (63.32 ± 4.14%), *j*_HCOOH_ (19.12 ± 3.21 mA cm^−2^) and *r*_HCOOH_ (990.83 ± 66.35 μmol m^−2^ s^−1^) obtained from the SGDE (Sn size: 100 nm; loading amount: 2.6 mg cm^−2^) are 13%, 44% and 44% lower than that obtained from the ESGDE-90 (*f*_HCOOH_: 73.01 ± 3.42%; *j*_HCOOH_: 34.21 ± 1.14 mA cm^−2^; *r*_HCOOH_: 1772.81 ± 59.08 μmol m^−2^ s^−1^), respectively. It indicates that the ESGDE-90 indeed enhance efficiency of ERCF, mainly owing to the enhancement in the reactants transfer and three-dimensional reaction zone.

Performance comparison of Sn-based GDE for ERCF reported in recent years are listed in Table S3 (see supplementary information). The *f*_HCOOH_, *j*_HCOOH_ and *r*_HCOOH_ obtained from the ESGDE-90 are somewhat higher than those obtained from Sn-based GDE in current studies under similar conditions. Recently, Irtem *et al*. electrodeposited Sn catalyst on a carbon fiber GDL and obtained a *f*_HCOOH_ of 71%, a *j*_HCOOH_ of 8.3 mA cm^−2^ and a *r*_HCOOH_ of 400 μmol m^−2^ s^−1^ ^39^. The *f*_HCOOH_ is almost the same as that of the ESGDE-90 while the *j*_HCOOH_ and the *r*_HCOOH_ are 3.1 times and 3.4 time lower than those of the latter, respectively. To clarify the different results, a Sn-based GDE was made by electrodepositing Sn directly onto the GDL of the SGDE with electrodeposition time of 90 s for ERCF. The *f*_HCOOH_ (55.93 ± 1.11%), *j*_HCOOH_ (21.54 ± 4.14 mA cm^−2^) and *r*_HCOOH_ (1116.24 ± 214.54 μmol m^−2^ s^−1^) obtained from this Sn GDE were 23%, 37% and 37% lower than those obtained from the ESGDE-90, respectively. Taking into account the composition and structure difference between this Sn-based GDE and the ESGDE-90, it can be inferred that the enhanced efficiency of ERCF mainly results from the active CL consisting of carbon black and Nafion, which enhances reactants transfer and three-dimensional reaction zone for ERCF.

**Figure 8:**
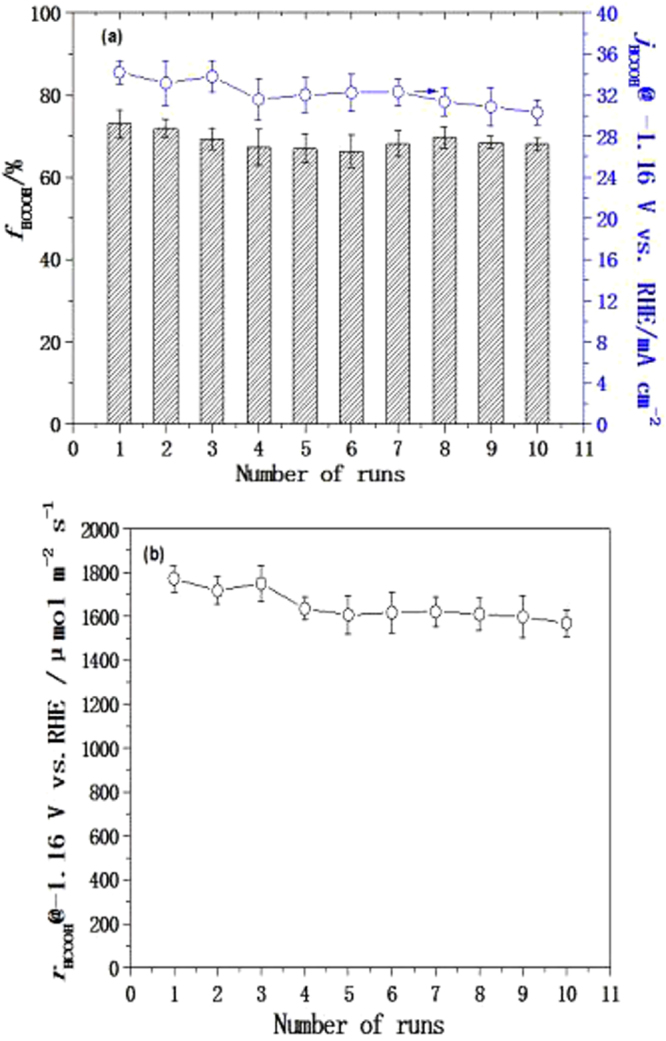
The *f*_HCOOH_, *j*_HCOOH_ and *r*_HCOOH_ as functions of the number of runs for ERCF at −1.16 V vs. RHE in 0.5 M CO_2_-saturated KHCO_3_ solution (pH = 7.47) at the ESGDE-90. Error bars based on three duplicate measurements for each test.

The stability of the GDE is critical for its industrial application in ERCF^4,25,29^. It has been reported previously that the catalysts could be gradually peeled off from the GDL (i.e. carbon paper), which is a common phenomenon observed in GDE systems, leading to a rapid decrease in the performance^4,13,24,25,40^. In order to examine the stability of the ESGDE-90, 10 successive runs for ERCF were carried out at the electrolytic potential of −1.16 V vs. RHE (Fig. 8a and b). It can be seen that just a slight decline occurred in the *f*_HCOOH_, *j*_HCOOH_ and *r*_HCOOH_ as the number of run increased. After a 10-time continuous run, the *f*_HCOOH_, *j*_HCOOH_ and *r*_HCOOH_ were decreased by 11% (from 73.01 ± 3.42% to 65.11 ± 1.50%), 11% (from 34.21 ± 1.14 mA cm^−2^ to 30.29 ± 1.18 mA cm^−2^) and 11% (from 1772.81 ± 59.08 μmol m^−2^ s^−1^ to 1569.67 ± 61.15 μmol m^−2^ s^−1^), respectively. Moreover, the loss of the catalyst particles is not obvious (weight loss: 1.05%, see Table S4 in the supplementary information) after a 10-time continuous ERCF test. These results indicate that the ESGDE-90 exhibits high stability in ERCF.

In short, the excellent electrochemical performance of the ESGDE-90 holds great promise for developing ERCF further, especially when considering the low fabrication cost (ca. 40 $ m^−2^). Further improvement of the dispersion degree of the Sn catalysts on the ESGDE-90 may be necessary to increase the efficiency of ERCF, allowing an economical viable process for CO_2_ mitigation and/or utilization.

## Methods

### Preparation of the ESGDEs

The ESGDE consists of a GDL and a CL. The GDL is made of carbon black (Cabot Corporation, USA), PTFE (Hesen Co. Ltd, China) and copper mesh (30 mesh, 100 μm wire diameter), which was fabricated according to our previous work^29^. The CL was prepared as follows: carbon black (Cabot Corporation, USA), Nafion ionomer (DuPont Corporation, USA) and deionized water were first mixing together to form ink, and then the ink was sprayed onto the GDL as CL support. The carbon black loading and Nafion fraction in the CL support was 1 mg cm^−2^ and 50 wt.%, respectively. Finally, Sn was electrodeposited on this CL support. The electrodeposition process was performed potentiostatically with a CHI600E electrochemical workstation (Shanghai Chenhua, China) in a self-assembled three-electrode cell. An Ag/AgCl (saturated KCl) electrode and a platinum sheet (1 cm^2^) were used as the reference electrode and counter electrode, respectively. The electrodeposited potential was −2 V vs. Ag/AgCl. The SnCl_2_ solution with a concentration of 0.05 M was used as the electrolyte. Sn was electrodeposited successively with different time of 30 s, 60 s, 90 s and 120 s, namely ESGDE-30, ESGDE-60, ESGDE-90 and ESGDE-120. The Sn loading amount of the ESGDEs were measured by weight difference.

### Characterizations and analytics

The prepared ESGDEs were characterized by SEM, TEM, XRD and XPS techniques. SEM images were taken by using S-4800 SEM microscope (Hitachi Limited) at an acceleration voltage of 30 kV. TEM images were obtained by using a JEOL-2100 TEM microscope with an acceleration voltage of 200 kV. XRD patterns were acquired by using the Smart Lab (9 kW) Rigaku XRD diffractometer, with Cu-Ka radiation. The XPS measurements were performed with an electron spectrometer (ESCALAB 250Xi, Thermo Fisher Scientific Inc.).

The electrochemical measurements involving CV and EIS were performed with a CHI600E electrochemical workstation (Shanghai Chenhua, China) in a self-assembled three-compartment electrochemical cell, described as previously^29^. The working electrode was the prepared ESGDEs (3.14 cm^2^). The counter electrode and the reference electrode were the platinum sheet (1 cm^2^) and Ag/AgCl (saturated KCl) electrode, respectively. All the electrode potentials were converted to the reversible hydrogen electrode (RHE) scale using the following equation: E (vs.RHE) = E (vs.Ag/AgCl) + 0.1988 + 0.0591 V × pH^15^. The electrolyte was KHCO_3_ solution with a concentration of 0.5 M (pH = 8.31). CV experiment was performed form 0 V vs. Ag/AgCl to −2 V vs. Ag/AgCl at a scan rate of 0.1 V s^-1^. EIS experiment was carried out over a frequency range of 100 kHz to 0.1 Hz with an AC perturbation of 0.005 V. The applied potential was −1.2 V vs. Ag/AgCl. Before each measurement, N_2_ was bubbled into the electrolyte for 30 min for a baseline. After that, either N_2_ or CO_2_ was bubbled into the electrolyte for 30 min for the actual measurements. During the measurements, N_2_ or CO_2_ was continuously sparged in the gas chamber.

ERCF tests were performed under potentiostatic conditions at ~298 K in the same three-compartment electrochemical cell described above. The CO_2_ was continuously sparged in the gas chamber during the ERCF experiment with the flow rate of 30 mL min^−1^. The ERCF experiments were terminated when the total charge passed reached 100 C. The formic acid concentration in the catholyte was quantified by using an ion chromatography (ICS-1500, Dionex, USA) using an AS14 (4 × 250 mm) separation column. The mixed solution of NaHCO_3_ (0.8 mM) and Na_2_CO_3_ (4.5 mM) was used as the mobile phase at a flow rate of 1 mL min^−1^. The Faraday efficiency for the formation of formic acid (*f*_HCOOH_) was calculated according to equation (1) as follows:

1 $${f}_{{HCOOH}}=\frac{{n}_{{HCOOH}}\times n\times F}{{\int }_{0}^{t}Idt}$$

where *n*_HCOOH_ is the moles of produced formic acid; *n* represents the number of electrons required for the formation of one molecule of formic acid from CO_2_, whose value is 2 here; *F* is Faraday’s constant whose value is 96,485 C mol^−1^; and *I* is the current.

The current density (*j*_HCOOH_) for the formation of formic acid was expressed as the current used for forming formic acid divided by the geometric area of the electrode.

## Electronic supplementary material

Supplementary Information
