# Supplementary material for: Electrodeposition of tin on Nafion-bonded carbon black as an active catalyst layer for efficient electroreduction of CO2 to formic acid
Source: Sci Rep. 2017 Oct 20;7:13711. doi: 10.1038/s41598-017-14233-y (PMC5651907; doi:10.1038/s41598-017-14233-y)
Supplement: Supplementary file 1 — Supplementary Information [file 41598_2017_14233_MOESM1_ESM.pdf]

# - Supplementary Information-

Electrodeposition of tin on Nafion-bonded carbon black as an active catalyst layer for efficient electroreduction of CO_2 to formic acid

Qinian Wang [1], Xinqi Wang [1], Chao Wu [1], Yuanyuan Cheng [2]*, Qingye Sun [1] , Heng Dong [3]*, Hongbing Yu [3]

[1] College of Resources and Environmental Engineering, Anhui University, Hefei, Anhui, 230601, China

[2] College of Life Sciences, Anhui University, Hefei, Anhui, 230601, China

[3] College of Environmental Science and Engineering, Nankai University, Tianjin 300071, China

*Corresponding Authors;

Yuanyuan Cheng, Phone: (86)0551-3861441, Fax: (86)0551-3861441, E-mail: chengyy@ahu.edu.cn;

Heng Dong, Phone: (86)22-85358115, Fax: (86)22-85358110, E-mail: dongheng@nankai.edu.cn.

Supplementary Table S1. Sn loading amounts of the prepared ESGDEs.

Electrode	ESGDE-30	ESGDE-60	ESGDE-90	ESGDE-120
Loading amount (mg cm^{-2})	0.9	1.9	2.6	3.4

Supplementary Table S2. Rate of formic acid production for the prepared ESGDEs, E= -1.16 V vs. RHE.

Electrode	r^{m}_{HCOOH} (μmol m^{-2} s^{-1} mg^{-1})	r^{s}_{HCOOH} (μmol m^{-2} s^{-1} mg^{-1})	r^{q}_{HCOOH} (μmol m^{-2} s^{-1} c^{-1})
ESGDE-30	1147.56 ± 69.67	84.09 ± 11.9	10.33 ± 0.63
ESGDE-60	685.95 ± 70.91	36.64 ± 6.44	13.03 ± 1.34
ESGDE-90	681.85 ± 22.72	28.67 ± 1.27	17.73 ± 0.59
ESGDE-120	374.49 ± 33.68	24.63 ± 4.63	12.73 ± 1.15

Supplementary Table S3. Summary of the literatures on Sn-based GDEs for ERCF in recent years.

Electrode	Operating condition	f_{HCOOH} /%	j_{HCOOH} /mA cm^{-2}	r_{HCOOH}/ μmol m^{-2} s^{-1}	Refs
Sprayed Sn and Nafion on GDL[a]	0.5 M $KHCO_3$, −1.8 V vs. Ag/AgCl	72.99	13.45	696.51 [b]	[1]
Sprayed Sn and Nafion on carbon paper	0.5 M $NaHCO_3$, −1.6 V vs. NHE	70.0	18.9	979.48 [b]	[2]
Rolling Sn-loaded copper mesh on GDL[a]	0.5 M $KHCO_3$, −1.8 V vs. Ag/AgCl	78.6	17.43	901.59 [b]	[3]
Sprayed Sn and Nafion on carbon paper	0.1 M $KHCO_3$, −1.6 Vcell	64	2.75	142.51 [b]	[4]
Sprayed Sn and Nafion on carbon paper	0.5 M $KHCO_3$, −1.6 V vs. SCE	72	ca. 20	746.23 [b]	[5]
Electrodeposited Sn on carbon fiber	0.5 M $NaHCO_3$, -1.05 V vs. RHE	71	8.3	400	[6]
Electrodeposited Sn on carbon paper	water vapor −1.6 V vs. Ag/AgCl	16	2	103.64 [b]	[7]
Sprayed SnO_2 and Nafion on carbon paper	0.5 M $KHCO_3$, −1.7 V vs. SHE	62	ca. 8	414.57 [b]	[8]
Sprayed 20% SnO_2/MWCNT and Nafion on carbon paper	0.5 M $NaHCO_3$, −1.7 V vs. SCE	27.2	21.76	1127.64 [b]	[9]
ESGDE-90	0.5 M $KHCO_3$, -1.16 V vs. RHE	73.01 ± 3.42	34.21 ± 1.14	1772.81 ± 59.08	This work

[a] GDL: consisting of carbon black, PTFE and copper mesh.

[b] This value is not mentioned in the article but calculated from the current density and Faraday efficiency.

Supplementary Table S4. Electrode weight loss for ESGDE-90 after ERCF test.

Number of runs	Electrode weight (mg cm^{-2})	Weight loss (%)
0	66.87	0
1	66.78	0.13
5	66.54	0.49
10	66.17	1.05

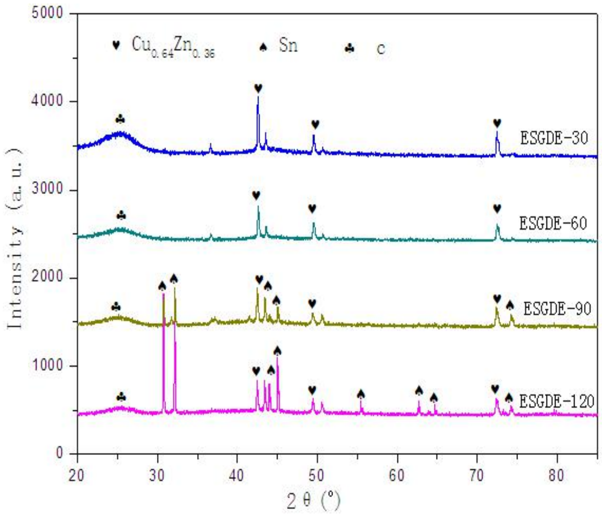

Supplementary Figure S1. XRD patterns of the ESGDEs. $Cu_{0.64}Zn_{0.36}$ is the major composition of the copper mesh in the ESGDEs.

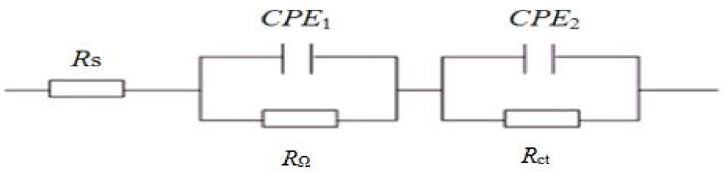

Supplementary Figure S2. Equivalent circuit used for the fitting of the impedance plots [1, 3]. R_s: solution resistance, R_{Ω}: Ohmic resistance, R_{ct}: charge-transfer resistance, CPE_1 and CPE_2: constant phase angle element.

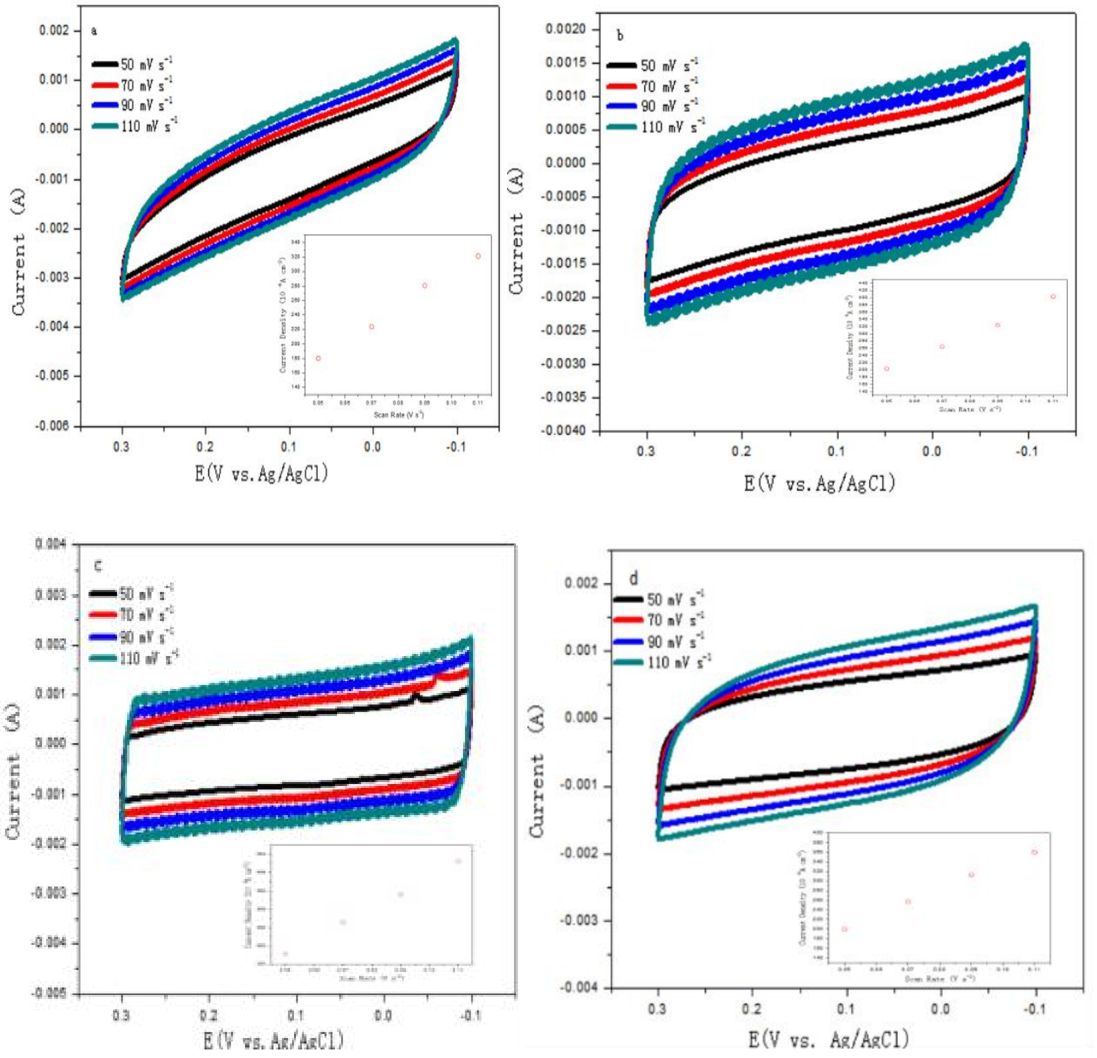

Supplementary Figure S3. Active surface areas derived from the capacitance of the ESGDE-30 (a), ESGDE-60 (b), ESGDE-90 (c) and ESGDE-120 (d), measured in N_2-saturated 0.1 M $KHCO_3$ solution. CVs were recorded for the working electrode in a potential range without Faraday process. Potential scan rates are marked in the figure. Insert shows a linear relationship between the current density and the scan rate.

References

1 Wang, Q., Dong, H. & Yu, H. Fabrication of a novel tin gas diffusion electrode for electrochemical reduction of carbon dioxide to formic acid. *RSC Adv.* **4**,

59970-59976, (2014).

2 Prakash, G. K. S., Viva, F. A. & Olah, G. A. Electrochemical reduction of CO_2 over Sn-Nafion® coated electrode for a fuel-cell-like device. *Journal of Power Sources* **223**, 68-73, (2013).

3 Wang, Q., Dong, H. & Yu, H. Development of rolling tin gas diffusion electrode for carbon dioxide electrochemical reduction to produce formate in aqueous electrolyte. *Journal of Power Sources* **271**, 278-284, (2014).

4 Wu, J., Risalvato, F. G., Ma, S. & Zhou, X.-D. Electrochemical reduction of carbon dioxide III. The role of oxide layer thickness on the performance of Sn electrode in a full electrochemical cell. *J. Mater. Chem. A* **2**, 1647-1651, (2014).

5 Wu, J., Sharma, P. P., Harris, B. H. & Zhou, X.-D. Electrochemical reduction of carbon dioxide: IV dependence of the Faradaic efficiency and current density on the microstructure and thickness of tin electrode. *Journal of Power Sources* **258**, 189-194, (2014).

6 Irtem, E. *et al.* Low-energy formate production from CO_2 electroreduction using electrodeposited tin on GDE. *J. Mater. Chem. A* **4**, 13582-13588, (2016).

7 Machunda, R. L., Ju, H. & Lee, J. Electrocatalytic reduction of CO_2 gas at Sn based gas diffusion electrode. *Current Applied Physics* **11**, 986-988, (2011).

8 Fu, Y. *et al.* Novel hierarchical SnO_2 microsphere catalyst coated on gas diffusion electrode for enhancing energy efficiency of CO_2 reduction to formate fuel. *Applied Energy* **175**, 536-544, (2016).

9 Bashir, S. *et al.* Electrocatalytic reduction of carbon dioxide on SnO_2/MWCNT

in aqueous electrolyte solution. *Journal of CO_2 Utilization* **16**, 346-353, (2016).
